# Sex-lethal regulates back-splicing and generation of the sex-differentially expressed circular RNAs

**DOI:** 10.1093/nar/gkad280

**Published:** 2023-04-18

**Authors:** Yu-Jie Fan, Zhan Ding, Yu Zhang, Ruibao Su, Jia-Le Yue, An-Min Liang, Qi-Wei Huang, Yan-Ran Meng, Muwang Li, Yuanchao Xue, Yong-Zhen Xu

**Affiliations:** The RNA Institute, State Key Laboratory of Virology, Hubei Key Laboratory of Cell Homeostasis, College of Life Sciences, TaiKang Center for Life and Medical Sciences, Wuhan University, Hubei430072, China; The RNA Institute, State Key Laboratory of Virology, Hubei Key Laboratory of Cell Homeostasis, College of Life Sciences, TaiKang Center for Life and Medical Sciences, Wuhan University, Hubei430072, China; The RNA Institute, State Key Laboratory of Virology, Hubei Key Laboratory of Cell Homeostasis, College of Life Sciences, TaiKang Center for Life and Medical Sciences, Wuhan University, Hubei430072, China; Institute of Biophysics, Chinese Academy of Sciences, Beijing 100101, China; The RNA Institute, State Key Laboratory of Virology, Hubei Key Laboratory of Cell Homeostasis, College of Life Sciences, TaiKang Center for Life and Medical Sciences, Wuhan University, Hubei430072, China; The RNA Institute, State Key Laboratory of Virology, Hubei Key Laboratory of Cell Homeostasis, College of Life Sciences, TaiKang Center for Life and Medical Sciences, Wuhan University, Hubei430072, China; The RNA Institute, State Key Laboratory of Virology, Hubei Key Laboratory of Cell Homeostasis, College of Life Sciences, TaiKang Center for Life and Medical Sciences, Wuhan University, Hubei430072, China; The RNA Institute, State Key Laboratory of Virology, Hubei Key Laboratory of Cell Homeostasis, College of Life Sciences, TaiKang Center for Life and Medical Sciences, Wuhan University, Hubei430072, China; College of Biotechnology, Jiangsu University of Science and Technology, Jiangsu 212018, China; Institute of Biophysics, Chinese Academy of Sciences, Beijing 100101, China; The RNA Institute, State Key Laboratory of Virology, Hubei Key Laboratory of Cell Homeostasis, College of Life Sciences, TaiKang Center for Life and Medical Sciences, Wuhan University, Hubei430072, China

## Abstract

Conversely to canonical splicing, back-splicing connects the upstream 3' splice site (SS) with a downstream 5'SS and generates exonic circular RNAs (circRNAs) that are widely identified and have regulatory functions in eukaryotic gene expression. However, sex-specific back-splicing in *Drosophila* has not been investigated and its regulation remains unclear. Here, we performed multiple RNA analyses of a variety sex-specific *Drosophila* samples and identified over ten thousand circular RNAs, in which hundreds are sex-differentially and -specifically back-spliced. Intriguingly, we found that expression of SXL, an RNA-binding protein encoded by *Sex-lethal* (*Sxl*), the master *Drosophila* sex-determination gene that is only spliced into functional proteins in females, promoted back-splicing of many female-differential circRNAs in the male S2 cells, whereas expression of a SXL mutant (SXL^RRM^) did not promote those events. Using a monoclonal antibody, we further obtained the transcriptome-wide RNA-binding sites of SXL through PAR-CLIP. After splicing assay of mini-genes with mutations in the SXL-binding sites, we revealed that SXL-binding on flanking exons and introns of pre-mRNAs facilitates back-splicing, whereas SXL-binding on the circRNA exons inhibits back-splicing. This study provides strong evidence that SXL has a regulatory role in back-splicing to generate sex-specific and -differential circRNAs, as well as in the initiation of sex-determination cascade through canonical forward-splicing.

## INTRODUCTION

Cellular RNA molecules are mostly linear with termini of 5'-phosphate and 3'-hydroxy group; however, circular RNAs have been broadly identified in the past decade, in which free termini are covalently ligated and form a closed loop (1,2). Circular RNAs extensively exist in eukaryotes from yeast (3,4), worms (5,6), flies (7,8), plants (9,10) and mice to humans (5,11). Eukaryotic circular RNAs are usually classified into two groups according to their biogenesis pathways: the circular intronic RNAs (ciRNAs) generated from intronic lariats that failed to be debranched after splicing, and the exonic circular RNAs (circRNAs) generated by back-splicing that covalently joins an upstream 3' splice site (3'SS) with a downstream 5'SS of exons, conversely to the canonical forward-splicing that joins an upstream 5'SS with a downstream 3'SS (1). CircRNAs with inside unspliced introns were also found and named as EIciRNAs, the exon-intron circRNAs (12).

In the past decade, biological methods and bioinformatic tools have been developed to identify circular RNAs based on their enrichments by RNase R digestion followed by next generation sequencing (1,5,13). RNase R is an enzyme that has strong 3′–5′ exoribonuclease activity and efficiently degrades linear RNAs but not circular RNAs (14). The first bioinformatic tool identifying circRNAs was Find_circ (5). As the algorithms improved, more efficient software has been designed, such as the CIRI (15) and CIRCexplorer (16). In principle, these tools recognize circRNAs through identification of the back-spliced junction (BSJ) reads, in which the CIRCexplorer supports analysis of back-based alternative splicing and *de novo* assembly of circRNAs.

Chemically, back-splicing is presumably catalyzed by the same machinery as canonical forward-splicing, including both the major and minor spliceosomes (17). *Cis*-elements in pre-mRNAs and *trans*-factors have shown critical roles in the regulation of back-splicing (Reviewed in 18,19). It has been reported that flanking intronic complementary sequences (ICSs) of the circularized exons such as the well-studied mammalian *Alu* repeats (20–23) and RBPs including QKI, NF90/NF110, FUS, MBL and HNRNPL (24–28) can facilitate back-splicing of their nearby exons. RNA helicases have been proposed to regulate back-splicing through unwinding the base-paired intronic complementary sequences (13,29). Similarly to canonical forward-splicing, alternative back-splicing has been extensively identified in mammals (16,30).

Much evidence has demonstrated that many highly abundant circular RNAs can act as microRNA and RNA-binding protein sponges and transcription regulators, or can be translated into peptides. Functions of circRNAs are correlated with their subcellular localizations. Most of the identified circRNAs are in the cytoplasm (31,32), which can function as microRNA sponges, such as *CDR1as* for harboring miR-7 in neuronal tissues, and its loss results in microRNA deregulation and defective brain function (5,33). Many circRNAs can be used as templates for protein translation (34–38). In the nucleus, circular RNAs regulate transcription, such as *ci-ankrd52* that associate with RNA polymerase II during transcription elongation (39), and EIciRNAs that interact with U1 snRNP and promote transcription of their parental genes (12). In addition, it has been found that circRNAs are enriched in exosome (40) and at high levels in body fluids due to their unusual stabilities, allowing for applications as biomarkers in cancer diagnosis and prognosis (41,42).

Previous studies have identified thousands of circular RNAs in *Drosophila*, which are mainly age-dependent and accumulate in neural systems (7,8), such as *Edis*, a brain-enriched circRNA from its cognate gene *Ect4*, regulates the innate immunity and neuronal development (43,44). One of the most abundant and well-studied *Drosophila* circRNAs is *circMbl*; its circularized exon 2 and flanking introns contain multiple strong and conserved sites for binding of MBL (Muscleblind), which allows for the self-regulation of *circMbl* biogenesis (27). Another regulatory example in *Drosophila* is the *Laccase2* circRNA, which is controlled by multiple hnRNPs and SR proteins (23). Unlike in mammals, flanking introns of most *Drosophila* circRNAs lack long-enough ICSs, and the biogenesis and regulation of the tissue- and sex-specific circRNAs in *Drosophila* have not been investigated.

In this study, we first performed multiple RNA-seq including sequencing of the mRNAs, the mRNA and ribosomal RNA-depleted with and without RNase R digestion from a variety of sex-specific *Drosophila* samples. We identified more than ten thousands circular RNAs, in which hundreds were sex-differentially expressed or back-spliced. We then found that many of the female-specificallly back-spliced circRNAs could be facilitated by SXL, an RBP encoded by *Sex-lethal* (*Sxl*), the master gene in the alternative splicing (AS)-regulated cascade of *Drosophila* sex-determination pathway. Through PAR-CLIP assay for identification of SXL-binding peaks in the female Kc cells and splicing assay of mutant mini-genes in the male S2 cells, we further demonstrated that SXL can bind to the circularized exon and their flanking sequences for regulation of the female-differentially back-spliced circRNAs.

## MATERIALS AND METHODS

### Fly strains, tissues and cell culture

The *WT* fly strain used in this study is an isogenic Canton-S strain (45); the *Sxl* mutant strains *Sxl ^f2^* (BDSC 4593) (46) and *Sxl ^M1, fΔ33^* (BDSC 58487) (47) were purchased from Bloomington Drosophila Stock Center. The heteroallelic *Sxl ^M1, fΔ33 / f2^*mutant was acquired from the cross between female virgins of *Sxl ^M1, fΔ33 / f2^/ Binsinscy* and the *Sxl ^f2^/Y* males. Sex-specific tissues for the *WT* strain were used for circRNA sequencing, and the body compartment from the *Sxl* mutants were applied for detection of circRNAs by RT-PCR. Culture and crosses of *D. melanogaster* were carried out on standard medium at 25°C. Adults of 24–30 hours post eclosion were dissected into head, body, ovary and testis (the entire inner male reproductive system, including testis, seminal vesicle, accessory gland, and ejaculatory duct). *Drosophila* S2 and Kc were cultured in the complete Schneider's Medium (GIBCO) that contains 10% FBS (GIBCO).

### Sequencing for circular and linear RNAs

Total RNAs from the six sex-specific samples were isolated by TRIzol (Sigma) and treated with DNase I (Takara), in which mRNAs (referred to as ‘PolyA’) were isolated using the Dynabeads^®^ mRNA Purification Kit (Ambion), and the rest of the RNAs were recovered for further depletion of rRNA (referred to as ‘R-’). In order to completely deplete rRNAs, 6 new probes targeting *Drosophila* specific 5S and 5.8S rRNAs mixed with previously reported 37 probes (48) were applied into the probe-pool from the Ambion RiboMinus kit (Supplementary Table S1). To obtain the enriched circular RNAs (referred to as ‘R+’), 2.5 μg of each R- sample was digested by 50 U of RNase R (Epicentre) at 37°C for 1 h followed by TRIzol purification and ethanol precipitation. All the RNA samples were subjected to the paired-end sequencing by Illumina HiSeq X10, and strand-specific libraries were applied to the R- and R + samples for sequencing.

### Data analysis of circular RNAs

After removal of adapter sequences from the raw reads by *cutadapt*_v1.18, the pair-end reads from the R- and R + samples were then merged using *PEAR*_v0.9.5, and the merged reads were discarded if < 30nt (49). The remaining merged reads and reads from the PolyA samples were mapped to the *Drosophila* genome (*dm6*) using *STAR*_v2.6.1a, where the parameter ‘-chim_segment_min’ was set to 10 and genes were annotated by a *Ensembl*_v6.88 database. Transcripts were assembled by *cufflinks*, and the CIRCexplorer2 (16) was used for construction of new transcript structure information to identify back-spliced junction (BSJ) from the fusion junction reads that were calculated by *STAR*. If the number of BSJ reads from a circular RNA in R+ sample is twice more than that of in the R- sample, the corresponding circRNA is considered reliable.

To determine a differentially-expressed circRNA between genders/tissues, difference of the BSJ reads between R + samples should be ≥ 4-fold, meanwhile differences of the BSJ reads between R- samples should be at the same direction. Considering low abundance of circRNAs, BSJ reads in R- samples were pre-normalized by the total sequencing reads with reduction of reads from mRNAs and rRNAs using a method derived from *DESeq2*. To determine a sex-specifically expressed circRNA, the BSJ reads in one gender's R+ sample should be at least 15 and not be detected in any of the opposite-gender samples. To determine a differentially back-spliced circRNA, difference of the BSJ reads between R+ samples should be ≥ 4-fold, meanwhile differences of the BSJ reads between R– samples should be at the same direction. Considering varied expression of the cognate gene between gender/tissues, the BSJ reads in R + samples were pre-normalized by TPM (transcript per million) of its cognate gene and should be ≥15 in at least one of the samples.

### RT-PCR, western blotting and co-immunoprecipitation

Reverse transcription was performed using 1 μg of total RNAs with random primers and RevertAid Reverse Transcriptase (Thermo), and the cDNAs were then amplified by Ex-Taq (TaKaRa). Divergent primers (Supplementary Table S1) were designed for PCR amplification of circular RNAs that were treated with or without RNase R. Primers for amplification of linear mRNAs from the cognate genes and *actin* and *βTub60D* genes were also listed in Supplementary Table S1.

For western blotting, protein samples were separated on a 10% SDS-PAGE and then transferred to a PVDF membrane (IPVH00010, Millipore). The monoclonal anti-SXL antibody M114 (DSHB), anti-6xHis antibody (H1029 sigma) and anti-α-tubulin antibody T6199 (Sigma) were used for visualization. For co-immunoprecipitation (co-IP), lysates from S2 cells transfected with blank pIZT, pIZT-SXL or pIZT-SXL^RRM^ were applied to the Protein G Dynabeads (Invitrogen) that bound with M114 antibody. The co-IPed proteins and RNAs were further analyzed by western blotting and RT-PCR.

### Vectors, plasmids, transformation and RNAi

Coding sequences (CDS) of the WT-SXL (FBtr0331250) and the mutant SXL^RRM^ with six alanine substitutions (N122A, Y123A, Q126A, R244A, V246A, and F248A), TRA, TRA2, DSX-F and DSX-M were cloned into the pIZT vector (Invitrogen). Plasmids with these CDSs were transfected into S2 cells by TransIT Transfection Reagent (Mirus Bio). The WT and mutant mini-genes for generating circRNAs were cloned into the pMT vector, which were then co-transfected with the blank pIZT or pIZT-SXL. Expression of the mini-genes was induced by 0.5 mM CuSO_4_ at the 12 h post-transfection, and cells were finally collected 48 h later for further isolation of RNAs and proteins (50).

The sequence of dsRNA that targets *Sxl* was chosen using SnapDragon (51), and knockdown of *Sxl* was performed by soaking the Kc cells with dsRNA (Supplementary Table S1) as described (52).

### Par-clip

PAR-CLIP in *Drosophila* Kc cells was performed based on two previous reports with modifications (53,54). Briefly, cells were grown overnight in the medium supplemented with 100 μM 4-thiouridine (4SU, Sigma) and then irradiated under 365 nm UV light for 400 mJ/cm^2^. Cells were then harvested and lysed in the NP40 lysis buffer [1× PBS (pH7.4) with 0.1% SDS, 0.5% deoxycholate and 0.5% NP40]. The cross-linked RNA-protein complexes were co-purified using anti-SXL antibody (M114, DSHB), which bound to the Protein G Dynabeads. Micrococcal nuclease (MNase, Thermo) was used for digestion of non-protected RNAs, which resulted in protein-bound short RNAs (50–100 nt) on beads. After ligation of 3′-RNA linker and 5′-labeling using γ-^32^P-ATP, the RNA-protein complexes were separated by SDS-PAGE and transferred to a nitrocellulose membrane, and the desired complexes on the membrane with radioactive signals at the range of 40–60 kDa (SXL is 42 kDa) were cut and digested by proteinase K (Thermo). The RNAs were then recovered by phenol/chloroform extraction and ethanol precipitation, followed by reverse transcription for construction of cDNA libraries and Illumina HiSeq X10 sequencing.

### Data analysis of PAR-CLIP

Raw reads were filtered by *CTK*_v1.1.3 (55), and their adaptor sequences were removed by *cutadapt*_v1.18 and extracted by *umi*_tools (56). Clean reads were then mapped to *Drosophila* genome (*dm6*) by *STAR* and annotated by *Ensembl*_v6.88, in which highly duplicated reads caused by PCR were removed by *umi*_tools. *OmniCLIP* was then applied for identification of the SXL-binding peaks (57), and *CTK*_v1.1.3 was used for searching CIMS with T > C mutation. Final significant SXL-binding peaks were defined if they match either (i) SXL_signal_/IgG_signal_ ≥ 10 (*P*-value < 0.0001) and T > C CIMS (FDR ≤ 0.3), or (ii) SXL_signal_/IgG_signal_ ≥ 100 (*P*-value < 0.0001) despite T > C CIMS.

### Construction of mini-genes that express circRNAs

Mini-genes for expression of circRNAs were constructed into the pIZT vector and transfected into S2 cells. For expression of *dmc_579*, the cloned fragment ranges from exons 1 to 5 of the cognate gene *crc* (CG8669), in which the middle part (3918 nt) of intron 3 without SXL-binding site was removed due to length limitation of cloning, and a trinucleotide (CGA) was inserted into the middle of exon 4 (position 199) to distinguish the exogenous- and endogenous-products. Similar strategies were used for cloning mini-genes that expressing *dmc_7247*. For construction of mutants of these mini-genes, the multiple Ts in the SXL-binding peaks were substituted by As. For location details and used primers see Figure 6 and Supplementary Table S1.

## RESULTS

### Identification of circular RNAs from multiple sex-specific samples

**Figure 1.**
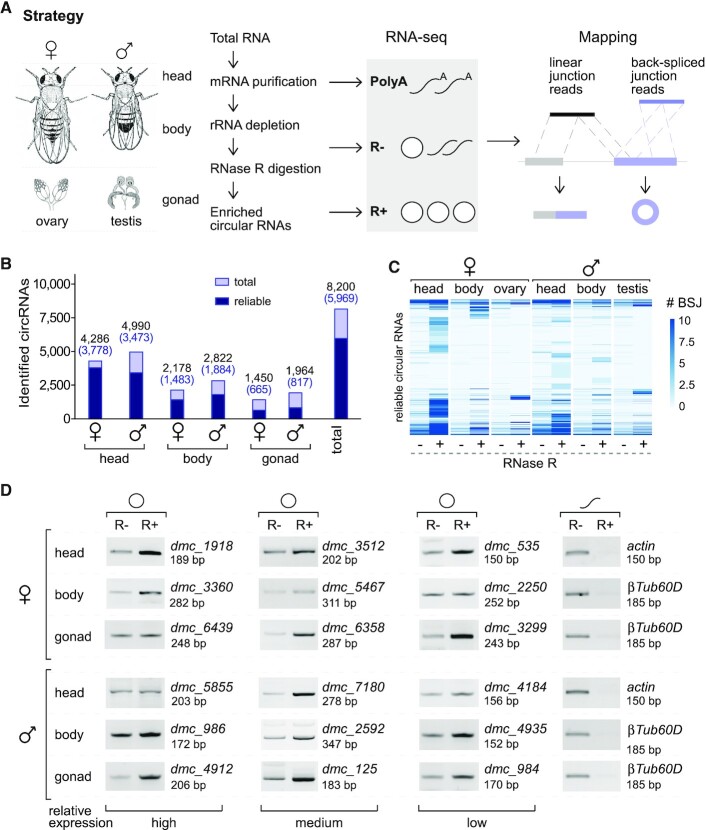
Identification of circular RNAs from *Drosophila* sex-specific samples through multiple next generation RNA sequencing. (**A**) Strategy utilized for sampling of fly adults (head, body and gonad from the females and males), construction of cDNA libraries from the fractionated RNAs, and mapping of reads from the linear and circular RNA products. (**B**) Statistics of the identified exonic circular RNAs in the six sex-specific samples. Light blue, total circRNAs; dark blue, reliable circRNAs. (**C**) Heatmap of the reliable circular RNAs in the sex-specific samples with and without RNase R digestion. Levels of circular RNAs were evaluated by their normalized back-spliced junction reads (BSJ). CircRNAs, exonic circular RNAs generated by back-splicing; ciRNAs, intronic circular RNAs generated from intronic lariats. (**D**) Validation of the identified circRNAs by RT-PCR. CircRNAs were clustered into high, medium and low three groups according their expression levels. Divergent primers were designed for amplification of the circRNAs (O) that were treated with RNase R (R+) or without (R–). Linear mRNA products (∽) from *actin* and *βTub60D* were used as controls. Sizes of all PCR products are indicated.

To investigate back-splicing in *Drosophila*, we sequenced a variety of sex-specific samples, including head, body and gonads from adults of both females and males (Figure 1A, left). Three RNA fractionations from each sample, including the mRNAs (PolyA), mRNA/rRNA-depleted RNAs (R–) and the enriched circular RNAs (R+, the R– fractions treated with RNase R), were purified and sequenced (Figure 1A middle). We obtained more than 500 G raw data and used the reversely-ligated BSJ reads to identify circular RNAs (Figure 1A right & Supplementary Table S2). In total, we identified 10075 circular RNAs (Supplementary Figure S1A and Supplementary Table S3), in which 8200 are circRNAs from the back-spliced exons (Figure 1B) and 1875 are ciRNAs from the intron lariats by canonical forward-splicing (Supplementary Figure S1B). In comparison to the CIRCpedia, 4850 circular RNAs we obtained are novel (Supplementary Figure S1C and Supplementary Table S3). To avoid false identification, we defined a reliable circular RNA as at least two-fold enriched after RNase R digestion; this gave 5969 reliable circRNAs and 1337 reliable ciRNAs (Figure 1B and Supplementary Figure S1B), in which 3932 are novels (Supplementary Figure S1C and Supplementary Table S3).

More than 60% of the reliable circRNAs were from the heads, while much less were from the bodies or gonads (Figure 1B and Supplementary Table S4), and many circRNAs exhibited higher expression in heads (Figure 1C); this is consistent with findings that the brain and neural systems are active in alternative splicing and have more circRNAs in the fruit fly and mammals (58–60). To validate, 18 reliable circRNAs from three expression groups (high, medium and low, see Supplementary Table S4) were randomly selected and confirmed by a process including RNase R digestion, RT-PCR with divergent primers and Sanger sequencing. These 18 circRNAs were significantly enriched after RNase R digestion, while the linear mRNAs from *actin* and *βTub60D* were totally digested (Figure 1D, cf. R+ to R–), demonstrating that the above bioinformatics analyses are reliable.

**Figure 2.**
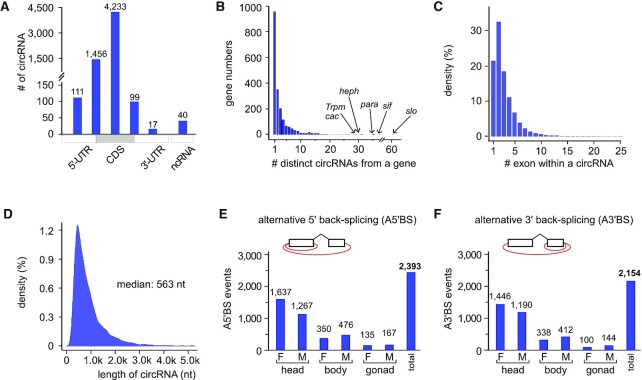
Characterization of *Drosophila* circRNAs. (**A**) Statistics of gene regions that originate circRNAs. (**B**) Numbers of the reliable circRNAs in a gene. Arrows show the genes that produce a large number of reliable circRNAs. (**C**) Statistics of exon numbers in a reliable circRNA. Most of circRNAs contains no more than 5 exons. (**D**) Statistics of the circRNA length. The median length is 563 nt. (**E**) Analysis of the alternative 5' back-splicing of the reliable circRNAs in *Drosophila*. (**F**) Analysis of the alternative 3' back-splicing of the reliable circRNAs. F, female; M, male. All the identified alternative back-splicing events are listed in Supplementary Table S5.

The reliable circRNAs were from 1980 *Drosophila* genes, in which <1% were from non-coding genes and the majority were from the middle exons of protein coding genes (Figure 2A). Nearly half of the cognate genes produced one circRNA isoform, while others produced multiples, such as genes *heph* (ortholog to human PTBP1), *sif* (ortholog to human TIAM1), *para* (ortholog to human SCN1A) and *slo* (ortholog to human KCNMA1) that can generate >30 different circRNAs (Figure 2B and Supplementary Table S5). Over two-thirds of the reliable circRNAs contained at least two exons, suggesting that occurrence of the inside canonical forward-splicing contributes to the generation of long circRNAs (Figure 2C). The majority were shorter than 1000 nt, while the median length was 563 nt (Figure 2D), similar to previous findings in fly and mammals (23,61). Lastly, we found that alternative back-splicing (AltBS) is frequent in *Drosophila*. There were 2393 and 2154 AltBS events at the 5'SSs and 3'SSs respectively, in which more than 65% of the AltBS events occurred in heads and much less were in bodies and gonads (Figure 2E, F and Supplementary Table S5).

### Tissue- and sex-differentially expressed circRNAs

**Figure 3.**
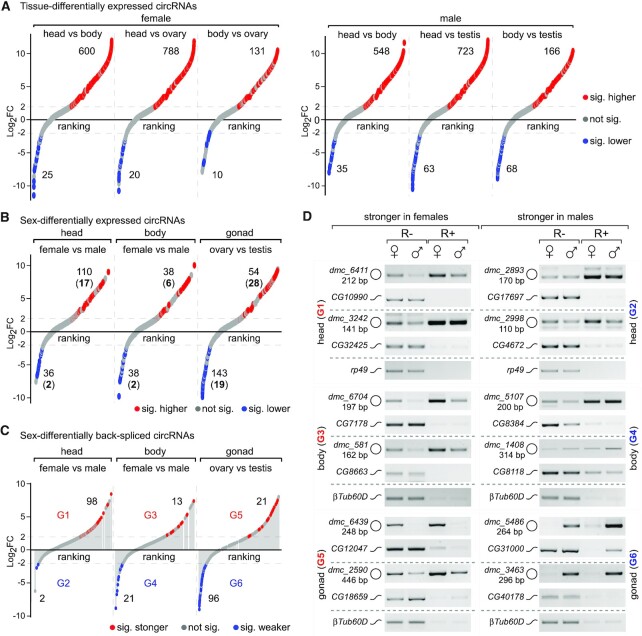
Identification of the sex-differentially expressed and back-spliced circRNAs. (**A**) Tissue-differential expression of circRNAs in the female (left) and male (right) *Drosophila* samples. (**B**) Sex-differential expression of circRNAs of the counterpart tissues between females and males. Expression levels of circRNAs were determined based on their BSJ reads. (**C**) Sex-differential back-splicing of circRNAs of the counterpart tissues between females and males. Back-splicing levels of circRNAs were determined based on their BSJ reads that were normalized by linear mRNAs of their cognate genes. Level changes ≥4-fold were defined as significant (sig.). The sex-differentially back-spliced circRNAs were classified into six groups (G1 to G6). All the x-axis are level ranking of the reliable circRNAs from low to high. (**D**) RT-PCR validation of the sex-differentially back-spliced circRNAs from the six groups. Each tested circRNA (O) was amplified by divergent primers from both the R+ and R– samples, and linear mRNA product (∽) from its cognate gene was also amplified. The mRNA levels of *rp49* and *βTub60D* were used as loading controls.

We then compared circRNA levels across the six sex-specific samples. In the female head, it has 600 and 788 circRNAs that were significantly expressed higher (≥ 4-fold) than those in the body and ovary respectively; whereas the body and ovary had much less, only 25 and 20 were significantly expressed higher than in the head, respectively (Figure 3A, left), and the similar patterns were observed in the male-specific samples (Figure 3A, right and Supplementary Table S6). Between two genders, there were 110 circRNAs significantly expressed higher in the female head than its counterpart, vice versa, 36 were significantly higher in the male head (Figure 3B left). The numbers were 54 and 143 when compared circRNAs between ovary and testis (Figure 3B right and Supplementary Table S6). In total, 40 and 21 circRNAs were expressed only in the female- and male-specific samples, respectively (Supplementary Figure S2A and Supplementary Table S6). These results indicate that many *Drosophila* circRNAs are tissue- and sex-differentially expressed.

### Sex-differentially back-spliced circRNAs

The level of a circRNA in cells depends on cumulative effects of multiple steps during the RNA processing, such as transcription of its cognate gene, back-splicing of the circularized exons, localization and stability of the circRNAs. To address which were sex-differentially back-spliced, we normalized circRNA levels to expression of their cognate genes using the mRNA-seq (PolyA) data (Figure 3C and Supplementary Table S6) and found that back-splicing of 98 circRNAs was significantly stronger in the female head than that of in the male head (G1 in Figure 3C), and 2 circRNAs were significantly stronger in the male head (G2 in Figure 3C). In contrast, back-splicing of 96 circRNAs was significantly stronger in testis than in ovary; *vice versa*, 21 were stronger in ovary (G5 and G6 in Figure 3C).

To validate this finding, we picked twelve circRNAs (two from each group) and detected levels of both the circRNAs and linear mRNAs in the sex-specific samples. Except for the two in G2, which only has two circRNAs with fold-changes very close to the cutoff, the other ten circRNAs were confirmed, displaying a pattern of sex-differentially back-spliced activity (Figure 3D). For example, the circRNA *dmc_6439* in G5 was specifically back-spliced in ovary but not in testis, while the linear mRNAs from its cognate gene *CG12047* were at similar levels in both gonads; and the circRNA *dmc_3463* in G6 was specifically back-spliced in testis but not in ovary, while the linear mRNAs from its cognate gene *CG40178* were at similar levels (Figure 3D).

### SXL promotes the generation of circRNAs

In total, there were 235 circRNAs which back-splicing activities were significantly different between the females and males (Supplementary Figure S2B). We then focused on factors in the sex-determination pathway to address whether they regulate the back-splicing activity. The sex-determination in *Drosophila* is somatic and controlled by a cascade of alternatively spliced genes including *Sex-lethal* (*Sxl*), *transformer* (*tra*) and *doublesex* (*dsx*) (62), in which *Sxl* and *tra* encode functional RNA-binding proteins SXL and TRA, respectively, in females but not in males, the *dsx* gene encodes transcription factors that differ in their C-termini, DSX-F in females and DSX-M in males (63).

**Figure 4.**
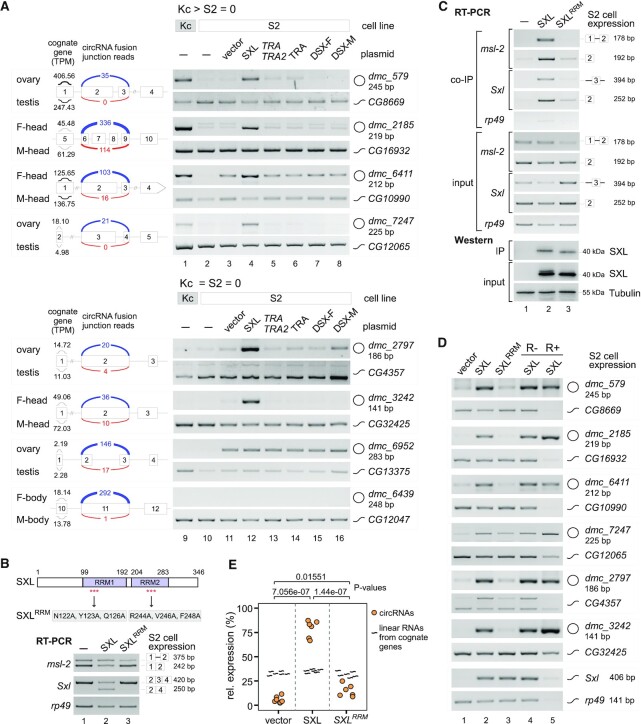
The RNA-binding protein SXL regulates back-splicing of the female-specific circRNAs. (**A**) Expression of SXL, but not other sex-determination factors, in the male S2 cells promotes the female-specifically back-spliced circRNAs that were identified from the *Drosophila* adult samples. Upper, circRNAs that were specifically expressed in Kc cells but not in S2 cells; lower, circRNAs that were neither detectable in Kc cells nor S2 cells. Expression levels of those factors are shown in Supplementary Figure S3A. BSJ reads are indicated as blue lines (female samples) and red lines (male samples), and the expression levels (TPM) of their cognate genes are indicated as black lines. (**B**) Unlike the WT-SXL, expression of the SXL^RRM^ mutant that has six alanine substitutions in S2 cells failed to promote AS of downstream genes. Mutation sites in SXL were designed according to a published structure (for details see Supplementary Figure S3C). (**C**) SXL co-IPed pre-mRNAs from its downstream genes, while the SXL^RRM^ mutant did not. (**D**) Expression of SXL promotes the female-specifically back-spliced circRNAs in S2 cells, while expression of the SXL^RRM^ mutant did not. (**E**) Quantification of the circRNAs in S2 cells with expression of SXLs. Data represents the mean ± SEM from six repeats, and the *P*-values were calculated using a two-sided *t*-test. *P < 0.1, **P < 0.01, ***P < 0.001.

We performed detection using two sex-specific cell lines, Kc from the female and S2 from the male (64). Showing by RT-PCR and western blotting, exogenous SXL, TRA, TRA2, DSX-F and DSX-M were expressed in S2 cells, which has no endogenous SXL, TRA and DSX-F (Supplementary Figure S3A and B). Among the 8 tested circRNAs that were sex-differentially back-spliced in the previous *Drosophila* samples, 4 circRNAs (*dmc_579, _2185, _6411* and _*7247*) exhibited expression in the female Kc cells but did not in the male S2 cells, whereas the other 4 circRNAs (*dmc_2797, _3242, _6952* and _*6439*) could not be detected in either Kc or S2 (Figure 4A, cf. lanes 1–2 and 9–10). Interestingly, expression of the exogenous SXL but not TRA, TRA + TRA2, DSX-F or DSX-M in S2 cells dramatically improved back-splicing of all the first 4 circRNAs and two of the second 4 circRNAs (Figure 4A, cf. lanes 3–4 and 11–12). Meanwhile, linear mRNAs of the increased circRNAs’ cognate genes were decreased or not obviously changed in S2 cells, suggesting that the stimulated production of those six circRNAs were dependent on the improved back-splicing activity rather than on an increased transcription activity, and this is consistent with the previous finding that exon circularization and linear splicing could compete with each other (27).

To further confirm, we constructed SXL^RRM^, a SXL mutant that has 6-residue substitutions (N122A, Y123A and Q126A in RRM1, and R244A, V246A and F248A in RRM2), based on information from a crystal structure of the SXL-RNA complex (65), in which the 6 residues directly interact with the pre-mRNA substrate (Figure 4B, upper and Supplementary Figure S3C). Expression of SXL^RRM^ in S2 cells did not change AS of two known target genes, *msl-2* and *Sxl* itself (Figure 4B, lower), and the SXL^RRM^ mutant co-IPed much less pre-mRNA of the two genes (Figure 4C), demonstrating that this mutant has significantly decreased RNA-binding activity. Furthermore, the SXL^RRM^ mutant failed to facilitate back-splicing of all six tested circRNAs, whereas the WT-SXL did (Figure 4D). These results in cell lines are consistent with the above findings in the fly samples. We conclude that some of the sex-differentially back-spliced circRNAs are facilitated by SXL, and the effective RNA-binding activity of SXL is required for the facilitation of back-splicing.

We also asked whether the strongly back-spliced circRNAs in Kc cells could be inhibited by knockdown of SXL. However, none of the three tested circRNAs were decreased when the *Sxl* was knocked down by dsRNA-induced RNAi (Supplementary Figure S4A). On the other direction, we tried to address whether the male-specifically back-spliced circRNAs could be facilitated in Kc cells when SXL is knocked down, but there was no detectable difference in back-splicing (Supplementary Figure S4B). These data imply that knockdown was insufficient to abolish the SXL activity by the RNAi approach in Kc cells. We then further tested effects in the *Sxl* down-regulated mutant strains using a heteroallelic mutant *Sxl ^M1, fΔ33 / f2^* strain that was generated from the cross between *Sxl ^M1, fΔ33^* and *Sxl ^f2^* (46,47). In the females of the heteroallelic strain, female-specific isoform of *Sxl* was abolished, and the production of three tested female-differentially spliced circRNAs were significantly decreased (Supplementary Figure S4D). These results provide *in vivo* evidence that loss of SXL affects generation of the sex-differentially spliced circRNAs.

### Identification of SXL-binding sites by PAR-CLIP

**Figure 5.**
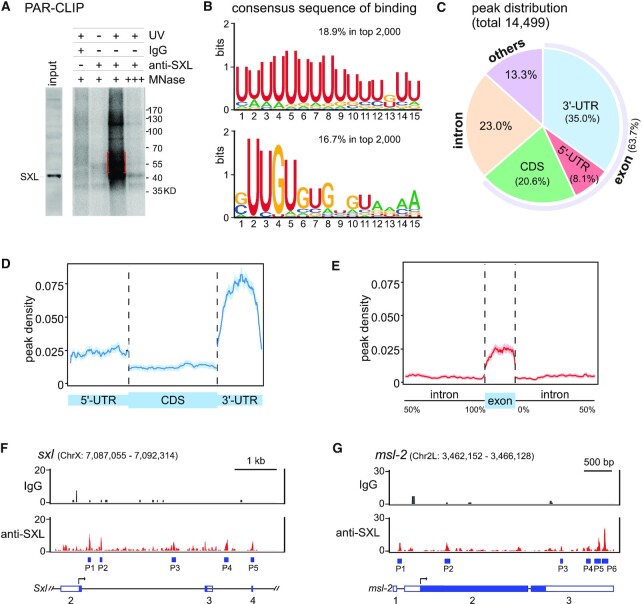
Distribution of the transcriptome-wide SXL-binding peaks. (**A**) Visualization of the ^32^P-labeled SXL-RNA adducts after MNase digestion and 3′-linker ligation, which is one step of the SXL PAR-CLIP in Kc cells. No UV-crosslinking, immunoprecipitation by IgG and over-digestion by MNase were used as negative controls. Region for further RNA extraction is indicated in a red rectangle. (**B**) The top two consensus RNA-binding motifs for SXL. (**C**) Distribution of SXL-binding peaks in the *Drosophila* genomic regions. (**D**) Comparison of the SXL-binding peak densities between regions of the 5'-UTR, CDS and 3'-UTR from coding genes. (**E**) Comparison of the SXL-binding peak densities between the intronic and exonic regions. (**F**) Identified SXL-binding peaks on the *Sxl* pre-mRNA. (**G**) Identified SXL-binding peaks on the *msl-2* pre-mRNA. Blue rectangles, positions of the SXL-binding peaks.

To address how the SXL-binding on pre-mRNA facilitates back-splicing and results in the generation of sex-specific circRNAs, we then performed PAR-CLIP to identify transcriptome-wide RNA-binding sites of SXL. After cross-linking, co-IP and RNA-seq of the SXL-bound RNA fragments in Kc cells (Figure 5A and Supplementary Figure S5A), we obtained 14499 SXL-binding peaks in total (Supplementary Figure S5B & Supplementary Table S7). Using software MEME-ChIP (66), two consensus RNA motifs for SXL-binding were identified, a U-rich and a GUUGU-rich motif (Figure 5B). The U-rich motif is consistent with a previous report using the SELEX method (67). However, the GUUGU-rich motif is a novel consensus motif, although its similar sequence was appeared in the identified SXL-binding site in the polypyrimidine tract (PPyT) of the *tra* pre-mRNA (65,68).

Among the SXL-binding peaks, 63.7% were from exonic regions, in which 8.1%, 20.6% and 35% were located in the 5'-UTRs, CDSs, and 3'-UTRs, respectively, and 23.0% were from intronic regions (Figure 5C). The highest density of SXL-binding in the 3'-UTRs implies that SXL may play critical roles in regulation of 3'-end cleavage/polyadenylation and translation (Figure 5D). The peak density was higher in exons than in introns (Figure 5E); however, SXL-binding in intronic regions was still significant if considering its low abundancy and stability in cells.

We did not obtain SXL-binding peaks on the *tra* pre-mRNA, which might be due to low level of the pre-mRNA in Kc cells. However, we obtained strong SXL-binding peaks on the other two known downstream targets. First, there were 5 peaks on the *Sxl* itself pre-mRNA, 3 were at intron 2 and the other 2 were at intron 3 and exon 4, respectively (Figure 5F); this is consistent with previous studies that SXL binds to intron 2 for self-splicing regulation and generates the *Sxl* mRNA in females (69). Second, there were 6 SXL-binding peaks on the *msl-2* pre-mRNA (Figure 5G), in which the peak P1 at intron 1 was near the two previously identified *cis*-elements that are silencers for splicing inhibition of the *msl-2* mRNA in females but not in males (70). In addition, 4 peaks (P3–P6) on the 3'-UTR of *msl-2* are consistent with studies that SXL inhibits translation of *msl-2* through its binding to the 3'-UTR (71,72). These data demonstrate that our PAR-CLIP data are reliable and useful for further mechanistic analysis.

### SXL-binding on flanking regions facilitates back-splicing, whereas binding on the back-spliced exons inhibits back-splicing

**Figure 6.**
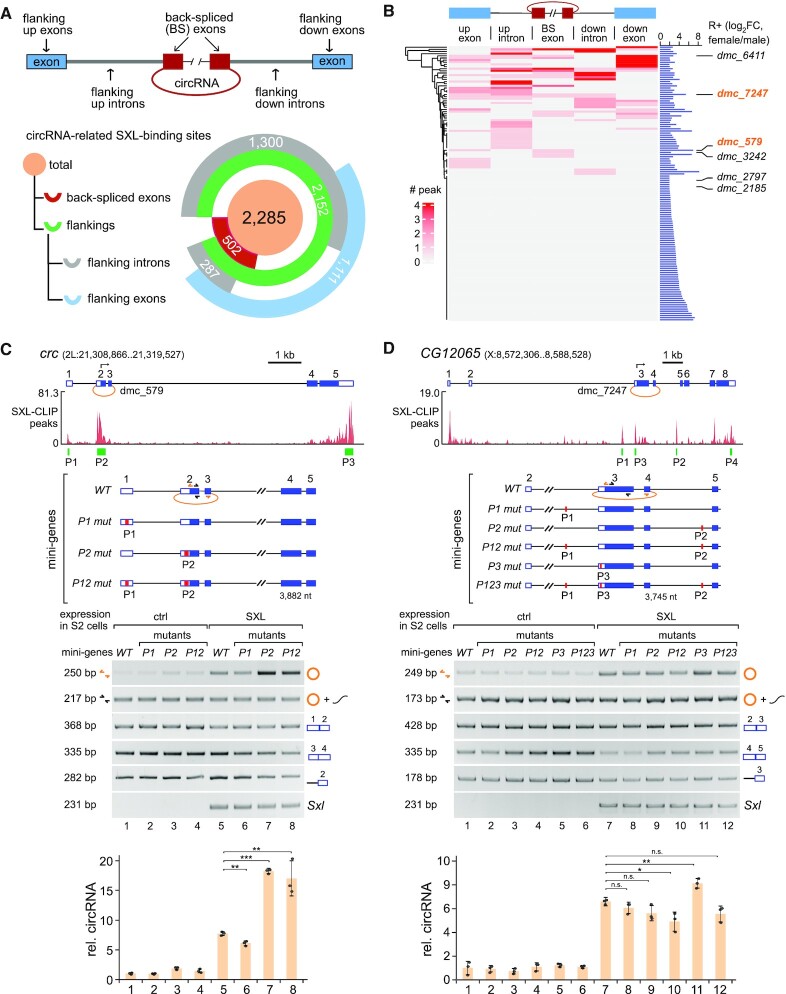
Binding of SXL regulates the sex-specifically back-spliced circRNAs. (**A**) Distribution of SXL-binding peaks in the circRNA-related regions. CircRNA-related regions are divided into the back-spliced exons, flanking introns and flanking exons. (**B**) Heatmap for SXL-binding peaks in the related regions of the 129 female-differentially back-spliced circRNAs. Six previously tested circRNAs are indicated. Distribution of SXL-binding peaks in the other circRNAs are presented in Supplementary Figure S6. (**C**) SXL regulates back-splicing of the circRNA *dmc_579* through binding to the upstream flanking exon and the back-spliced exon. (**D**) SXL regulates back-splicing of the circRNA *dmc_7247* through binding to the flanking introns and the back-spliced exons. Green rectangles, positions of the SXL-binding peaks; red rectangles, positions of the binding sites with T > A substitutions in the mini-gene mutants. Data represents the mean ± SEM from triplets, and the P-values were calculated using a two-sided *t*-test. *P < 0.1, **P < 0.01, ***P < 0.001.

We found 2285 reliable circRNAs that have SXL-binding peaks on the back-splicing related regions, including the back-spliced exons, flanking introns or flanking exons (Figure 6A). Among them, 502 (22.0%) circRNAs had peaks on their back-spliced exons, 1587 (69.5%) and 1111 (48.6%) circRNAs had peaks on their flanking introns and flanking exons respectively, showing a pattern that the SXL-binding peaks on the flanking regions are significantly enriched.

Further analyses revealed that 60 of the 129 female-differentially back-spliced circRNAs (G1, G2 and G3 in Figure 3C) had SXL-binding peaks on their back-splicing related regions, including four circRNAs (*dmc_579*, _*6411, _7247* and _*3242)*, whose back-splicing was facilitated by expression of SXL in S2 cells (Figure 6B). In contrast, 69 other circRNAs did not have SXL-binding peaks on their related regions. This result suggests that about half of the female-differentially back-spliced circRNAs may be facilitated by SXL-binding to their pre-mRNAs.

To address this hypothesis, we focused on previously confirmed two female-specifically back-spliced circRNAs (*dmc_579* and *dmc*_*7247*) and constructed their plasmid-borne *WT* and mutant mini-genes, in which the *WT* U-rich motifs in the SXL-binding peaks were replaced by A-rich sequences. First, there were two SXL-binding peaks related to the circRNA *dmc_579*, P1 on its flanking upstream exon and P2 on its back-spliced exon (Figure 6C). Similar to the previously tested endogenous *dmc_579* (Figure 4D), the *WT* mini-gene did not produce obvious *dmc_579* in S2 cells, but it was significantly promoted upon SXL-expression (Figure 6C cf. lanes 5 to 1). However, the P1 mutant mini-gene exhibited a decreased enhancement of *dmc_579*, whereas the P2 mutant exhibited an increased enhancement, and the P12 double-mutant showed mutually cancelled effects (Figure 6C). Meanwhile, the canonical spliced products (E1-E2) were at slightly lower levels upon expression of SXL.

Secondly, there were three SXL-binding peaks related to *dmc_7247*, P1 and P2 on the flanking upstream and downstream introns respectively, and P3 on its back-spliced exon (Figure 6D). Similarly, the level of *dmc_7247* from its *WT* mini-gene was promoted by SXL-expression in S2 cells. The enhancement was decreased when sequences of the P1 or P2 on the flanking introns were mutated, whereas the enhancement was increased when the P3 sequence on its back-spliced exon was mutated (Figure 6D, cf. lanes 8–11 to 7).

Taken together, results from splicing of the mini-genes and their mutants reveal that disruption of the SXL-binding on the flanking regions (either exon or intron) results in decreased back-splicing, whereas disruption of the SXL-binding on the back-spliced exon results in increased back-splicing.

## DISCUSSION

There are many biochemical layers that could control the production of a cellular circRNA. In this study, we identified thousands of reliable circRNAs from a number of sex-specific *Drosophila* samples and found that hundreds of them are sex-differentially and sex-specifically back-spliced. Focusing on the female-specifically back-spliced circRNAs, we demonstrate that the RBP SXL is a key regulator that binds to pre-mRNAs of cognate genes and controls the back-splicing activity in generation of many circRNAs.

**Figure 7.**
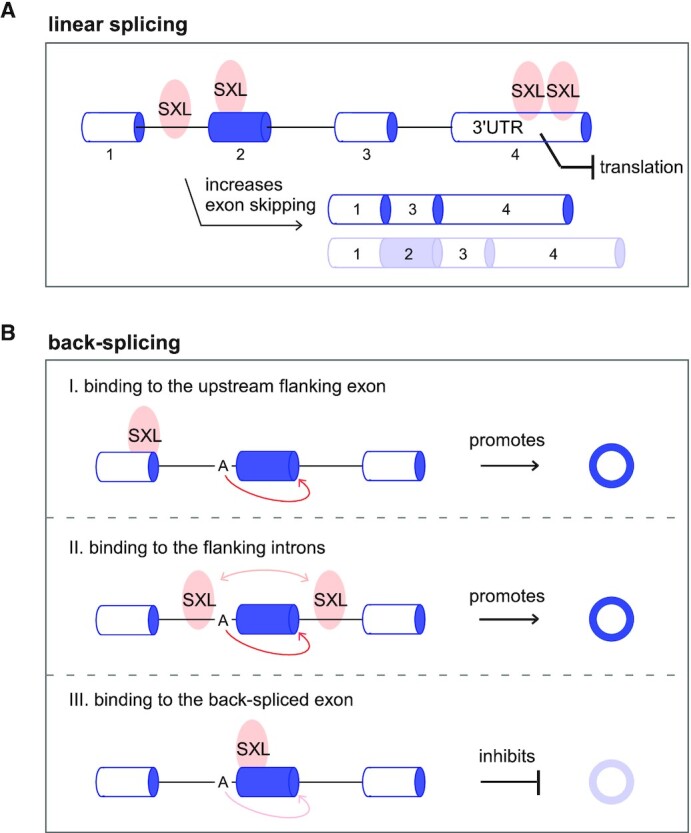
SXL regulates back-splicing and generation of sex-differentially expressed circRNAs. (**A**) SXL binds to introns and exons of the pre-mRNAs and often results in exon skipping under the way of canonical forward-splicing. Most of the SXL-binding peaks are enriched in the 3'-UTRs, implying a common translational control by SXL in *Drosophila* females. (**B**) SXL regulates back-splicing of female-specific circRNAs through three mechanistic modes. I) Binding of SXL to the upstream flanking exon inhibits canonical forward-splicing and promotes back-splicing; II) SXL binds to the flanking introns and promotes back-splicing, maybe functioning as a homodimer; III) SXL binds to the back-spliced exon and inhibits back-splicing.

Consistent with previous studies (69,70,73,74), our PAR-CLIP data demonstrate that SXL binds to introns and exons of its target genes, including the dosage compensation gene *msl-2* and *Sxl* itself, and result in skipped exons of their pre-mRNA splicing (Figure 7A). We also provide evidence that most of the SXL-binding peaks are particularly enriched in the 3'-UTRs of thousands of genes (Figure 7A), implying that SXL-binding on the 3'-UTR may be a common translational control for many genes in the female *Drosophila*, including the previously identified gene *msl-2* (71,72).

Besides controlling the fate of many linear mRNAs, in this study, we demonstrate that SXL can also regulate back-splicing of many female-specific and -differential circRNAs through three mechanistic modes. First, when SXL binds to the upstream flanking exon of a circRNA, it promotes the back-splicing due to inhibition of the canonical forward-splicing between the upstream exon and the circularized exon (Figure 7B, I). Second, when SXL is bound to the flanking introns of a circularized exon, it promotes the back-splicing activity that could be due to two reasons. One could be inhibition of the canonical forward-splicing between the circularized exon and the flanking exons. The other could be that SXL may function as a dimer (67) to bring the two splice sites closer for back-splicing and facilitate the production of circRNAs (Figure 7B, II), similar to the RNA-binding protein QKI that functions as a dimer and binds to the pre-mRNA to facilitate back-splicing (24). Third, when SXL binds to the back-spliced exon, it inhibits the back-splicing due to blocking the necessary splice sites for generation of circRNA (Figure 7B, III).

We notice that 69 out of the 129 female-differentially back-spliced circRNAs do not have SXL-binding peaks (Figure 6B); this could be due to either their expression levels being too low to be captured or that their back-splicing was modulated by other RBPs or *cis*-elements. Similarly, 61 out of the 112 male-differentially back-spliced circRNAs have SXL-binding sites (Supplementary Figure S6), suggesting that back-splicing of these circRNAs could be inhibited by SXL-binding in the females. It will be interesting to address their regulatory mechanisms in the future. It will also be important to know whether the identified 235 sex-differentially back-spliced circRNAs have functions in the *Drosophila* sexual development and how they contribute to the maturation of fly gonads and gametogenesis.

In addition, we obtained more than ten thousand RNA-binding sites of SXL transcriptome-wide in the female Kc cells through the PAR-CLIP approach; this will allow for future detail studies on new *Drosophila* genes whether their expression and splicing are regulated by SXL. It has been known that *sister-of-Sex-lethal* (*ssx*), a paralogue of *Sxl* that is not alternatively spliced between the females and males, has similarities in functions and RNA-binding properties compared to *Sxl* (75–77). In cooperation with SXL, SSX may play a secondary role in the SXL-regulated sex-specific back-splicing events.

## DATA AVAILABILITY

All NGS data have been deposited to NCBI under accession number GSE221102.

## Supplementary Material

gkad280_Supplemental_Files
