# Supplementary material for: Sex-lethal regulates back-splicing and generation of the sex-differentially expressed circular RNAs
Source: Nucleic Acids Res. 2023 Apr 18;51(10):5228–41. doi: 10.1093/nar/gkad280 (PMC10250224; doi:10.1093/nar/gkad280)
Supplement: gkad280_Supplemental_Files [file gkad280_supplemental_files.zip › Supplement_Figures.pdf]

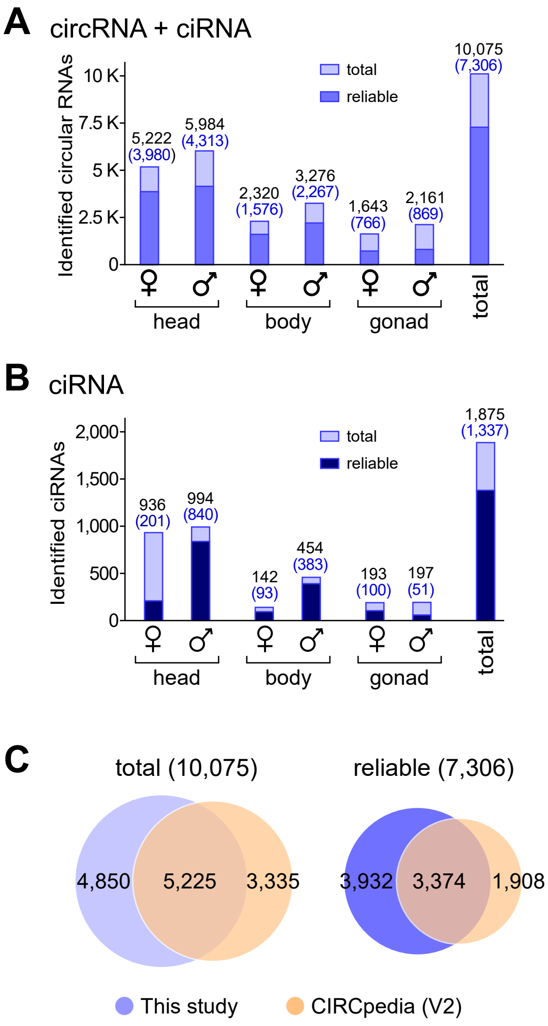

Figure S1. Identified circular RNAs in the sex-specific *Drosophila* samples.

(**A**) Statistics of all the identified circular RNAs, including circRNAs and ciRNAs, in the six sex-specific samples. (**B**) Statistics of the identified circular intronic RNAs (ciRNAs). Light blue, total circular RNAs; dark blue, reliable circular RNAs. (**C**) Comparison of the identified Drosophila circular RNAs between this study and the CIRCpedia v2 database. Left, the total detected circular RNAs; right, the reliable circular RNAs. CircRNA, exonic circular RNA generated by back-splicing; ciRNA, intronic circular RNA generated from intronic lariats.

Fan *et al*_Fig. S2

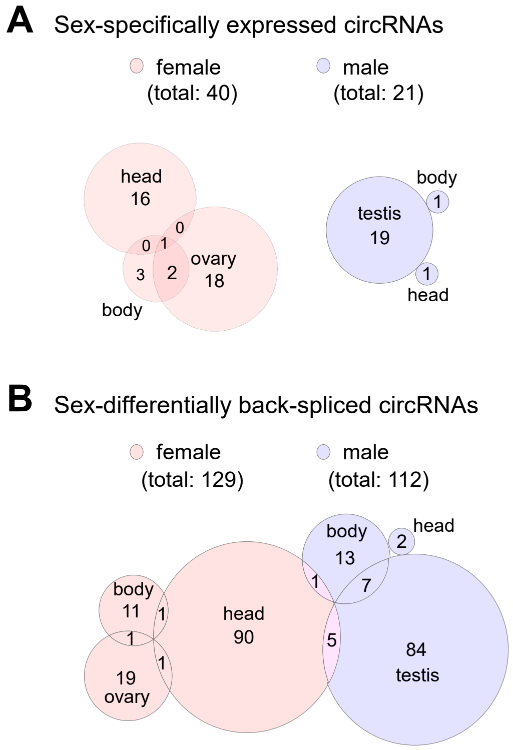

Figure S2. Statistics of the sex-specifically expressed circRNAs and sex-differentially back-spliced circRNAs in *Drosophila*.

(**A**) Statistics of the sex-specifically expressed circRNAs in the six *Drosophila* samples. (**B**) Statistics of the sex-differentially back-spliced circRNAs in the six sex-specific samples. Total numbers in each gender and their overlaps are indicated.

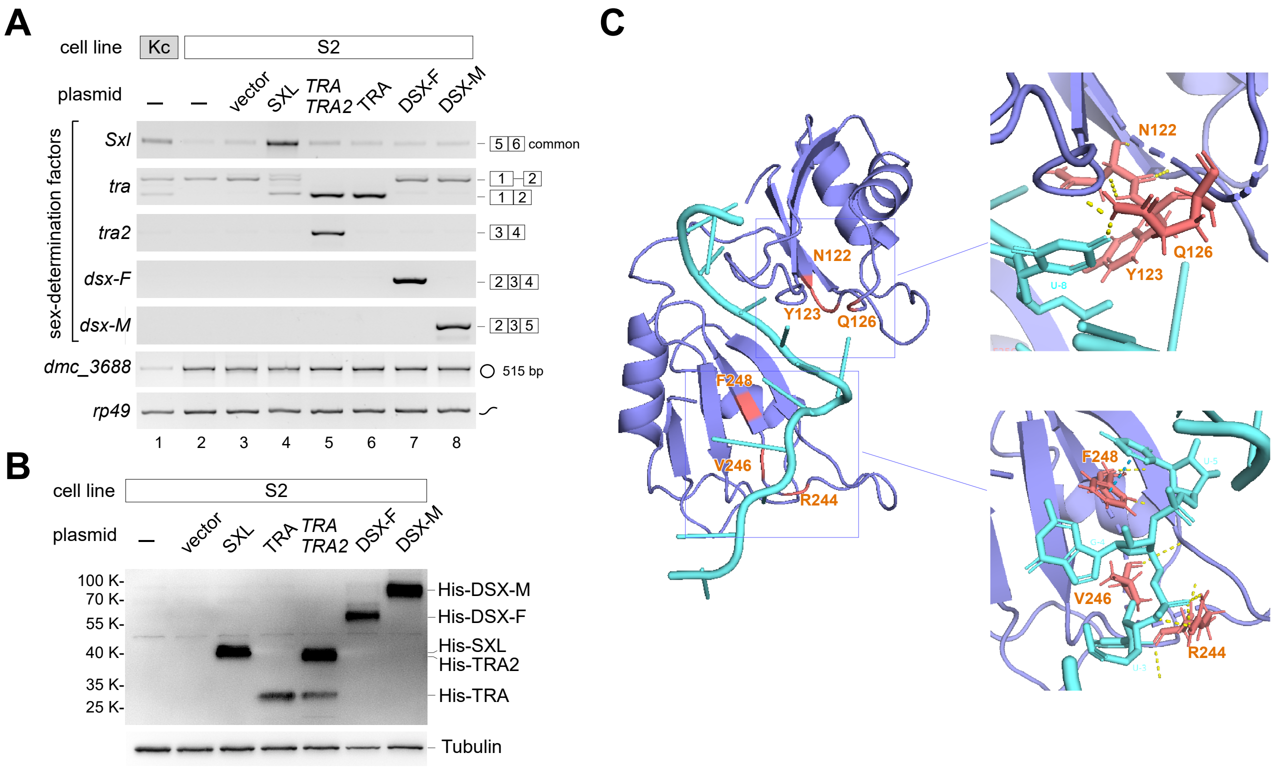

Figure S3. Expression of key factors from the *Drosophila* sex-determination pathway and the SXL^{RRM} mutant in the male S2 cells.

Detection of the expression levels of sex-determination factors in S2 cells by RT-PCR (**A**) and western blotting (**B**) for Figure 3A. The RNA levels of *dmc_3688* and *rp49,* and the protein levels of Tubulin were used as loading control. Expressed proteins were visualized by antibody against 6xHis. (**C**) Direct interactions between the RNA substrate and SXL residues. Left, six residues (brown) in SXL interact with the co-crystalized RNA substrate that was derived from the PPyT of *tra*; right, zoom on the regions around the six SXL residues that interact with the RNA. Blue, SXL; cyan, RNA substrate; brown, the RNA-interaction residues in SXL. Dashed lines: hydrogen bonds (yellow) and Pi-Pi interaction (blue). This figure is rendered from PDB ID: 1B7F (63) using PyMOL.

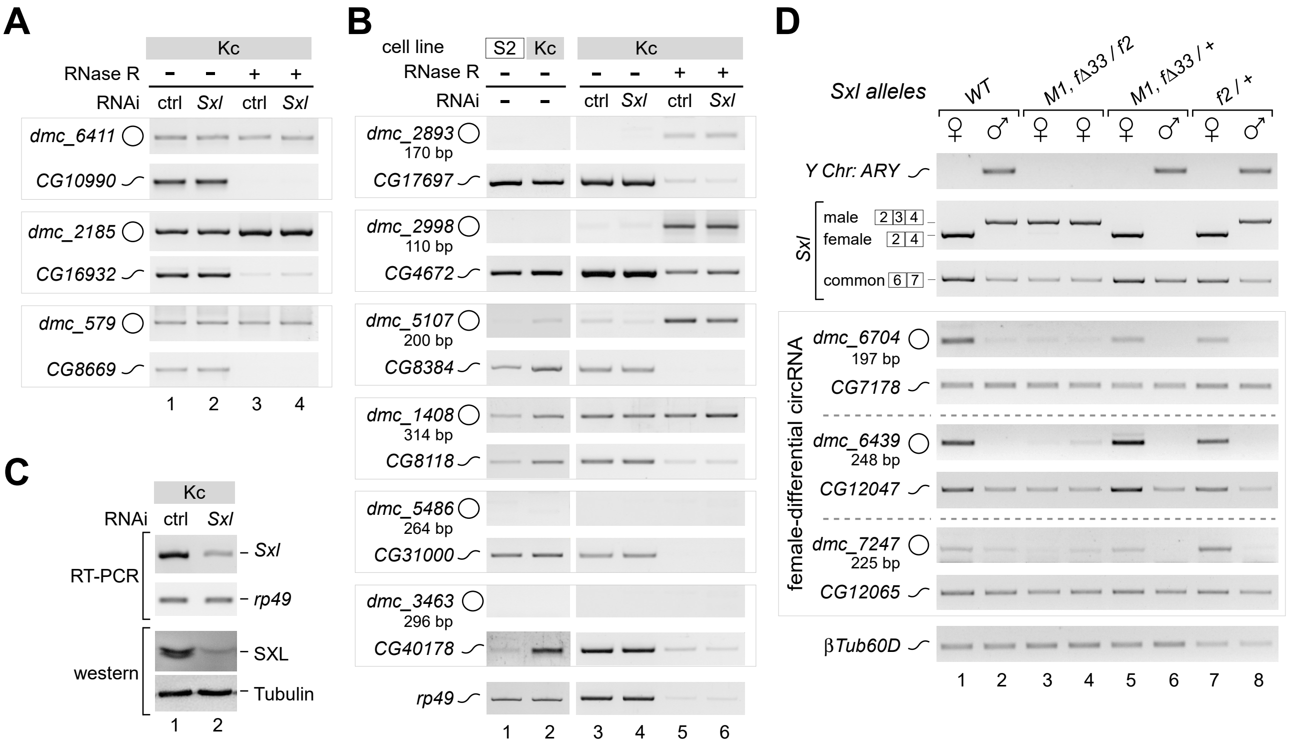

Figure S4. Knockdown of *Sxl* in *Drosophila* change the levels of sex-differentially back-spliced circRNAs.

(**A**) Three circRNAs significantly back-spliced in the female fly samples were not decreased when the *Sxl* was knockdown by dsRNA-induced RNAi in Kc cells. (**B**) Six circRNAs significantly back-spliced in the male fly samples were not increased when the *Sxl* was knockdown by RNAi in Kc cells. (**C**) *Sxl* was knocked down in Kc cells by the dsRNA-induced RNAi, which was confirmed by both RT-PCR and western blotting. (**D**) In the female bodies of $Sxl^{M1,\ f\Delta33\ /\ f2}$ mutant flies, *Sxl* is down-regulated and levels of the female-differenially spliced circRNAs are significantly decreased. The heteroallelic mutant $Sxl^{M1,\ f\Delta33\ /\ f2}$ was acquired from the cross between female virgins of $Sxl^{M1,\ f\Delta33}$ / *Binsinscy* (BDSC 58487) and Sxl^{f2}/*Y* (BDSC 4593). Genders of the fly adults were confirmed by expression of the Y chromosomal gene *ARY*. Sex-specific RNA isoforms of *Sxl* and their common regions were amplified using specific primers.

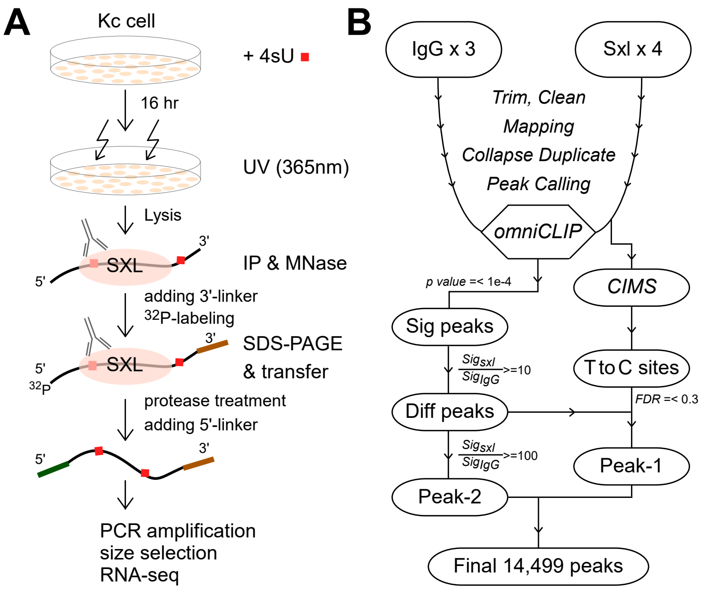

Figure S5. Strategy and pipeline for identification of transcriptome-wide SXL-binding using a PAR-CLIP approach and RNA-seq.
(**A**) Overview of the PAR-CLIP approach for identification of SXL's RNA-binding sites. (**B**) The processing pipeline for analysis of the CLIP-seq data.

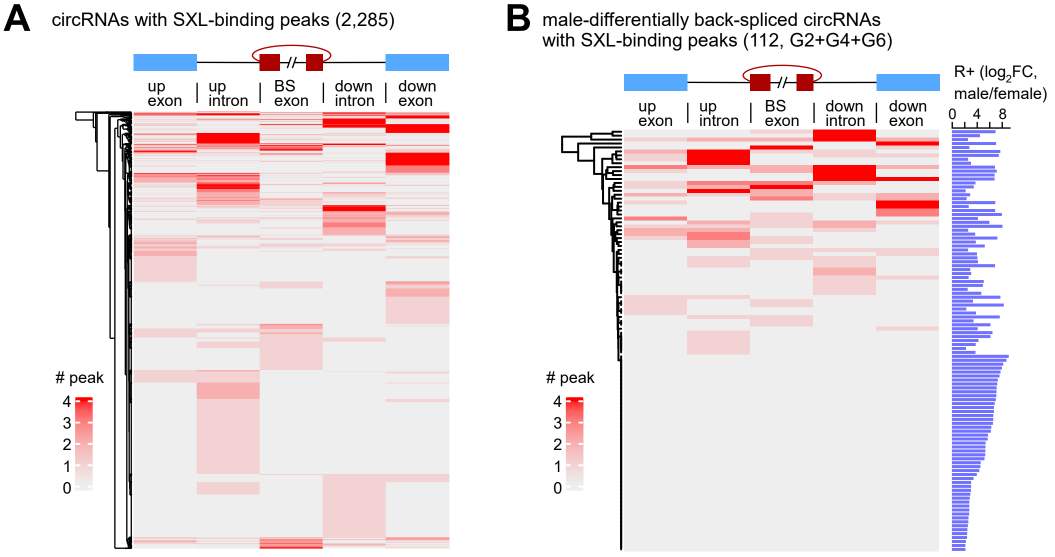

Figure S6. Heatmap for distribution of SXL-binding peaks in the related regions of *Drosophila* circRNAs.
(A) All the 2,285 circRNAs which related regions have SXL-binding peaks. (B) The male-differentially back-spliced circRNAs.
